# Comprehensive splicing graph analysis of alternative splicing patterns in chicken, compared to human and mouse

**DOI:** 10.1186/1471-2164-10-S1-S5

**Published:** 2009-07-07

**Authors:** Elsa Chacko, Shoba Ranganathan

**Affiliations:** 1 Department of Chemistry and Biomolecular Sciences, Macquarie University, Sydney, NSW 2109, Australia; 2 Department of Biochemistry, Yong Loo Lin School of Medicine, National University of Singapore, 8 Medical Drive, Singapore 117597

## Abstract

**Background:**

Alternative transcript diversity manifests itself as a prime cause of complexity in higher eukaryotes. Recently, transcript diversity studies have suggested that 60–80% of human genes are alternatively spliced. We have used a splicing pattern approach for the bioinformatics analysis of Alternative Splicing (AS) in chicken, human and mouse. Exons involved in splicing are subdivided into distinct and variant exons, based on the prevalence of the exons across the transcripts. Four possible permutations of these two different groups of exons were categorised as class I (distinct-variant), class II (distinct-variant), class III (variant-distinct) and class IV (variant-variant). This classification quantifies the variation in transcript diversity in the three species.

**Results:**

In all, 3901 chicken AS genes have been compared with 16,715 human and 16,491 mouse AS genes, with 23% of chicken genes being alternatively spliced, compared to 68% in humans and 57% in mice. To minimize any gene structure bias in the input data, comparative genome analysis has been carried out on the orthologous subset of AS genes for the three species. Gene-level analysis suggested that chicken genes show fewer AS events compared to human and mouse. An event-level analysis showed that the percentage of AS events in chicken is similar to that of human, which implies that a smaller number of chicken genes show greater transcript diversity. Overall, chicken genes were found to have fewer transcripts per gene and shorter introns than human and mouse genes.

**Conclusion:**

In chicken, the majority of genes generate only two or three isoforms, compared to almost eight in human and six in mouse. We observed that intron definition is expressed strongly when compared to exon definition for chicken genome, based on 3% intron retention in chicken, compared to 2% in human and mouse. Splicing patterns with variant exons account for 33% of AS chicken orthologous genes compared to 24% in human and 27% in mouse, providing a novel measure to describe the species-wise complexity due to alternative transcript diversity.

## Background

Alternative splicing (AS) is a fundamental mechanism that is believed to be a cause for protein diversity in higher eukaryotes. The introns in the pre-mRNA are removed in a process called splicing and the exons are coupled in varying combinations. This can change the composition of the primary transcript. A single gene can therefore generate a number of unique transcripts by combining exons and introns in different combinations, leading to the phenomenon of AS. Several recent studies estimated that >60% of human and mouse genes undergo alternative splicing [1-7].

It is critical to conduct an in-depth study on AS because it has been observed that the disruption of AS is associated with many diseases such as cardiovascular, cancer and neurodegenerative disorders [6]. Analyses have also shown that up to 15% of all point mutations causing human genetic disease result in an mRNA splicing defect [8], providing a link between AS events and inherited genetic diseases.

Alternative Splicing is an important mechanism that controls gene regulation and phenotypic complexity. This realization has resulted in several large-scale efforts to create bioinformatics resources on alternative transcripts and protein isoforms. However, previous analyses aiming to compare the quantum of alternative splicing between different organisms have provided conflicting results. Bork and co-workers [9] estimated that, in a large-scale expressed sequence tag (EST) analysis across distinct eukaryotes, all vertebrates and invertebrates showed a similar magnitude of alternative splicing with respect to both the number of genes affected and the number of variants per gene. But, according to the work done by Lee and co-workers [3], it has been shown that there is considerable variation in the amount of alternative splicing across organisms.

As more eukaryotic genomes are sequenced and annotated, several databases dedicated to AS are now available [1-7], leading to genome-wide computational analysis, reviewed by Lee and Wang [10]. Although these AS databases give an insight into the level of alternative splicing, they do not provide any visual representation and classification of the types of alternative splicing events occurring [10]. Moreover, as the number of transcripts per gene increases, it has become increasingly difficult to identify branch points and systematically analyse and classify AS events. Modrek and Lee [11] used directed acyclic graphs for EST analysis, with the genomic DNA sequence as reference. Pevzner and co-workers [12] were the first to use *de Bruijn *graphs to depict the transcripts alone, without referring to the genomic DNA sequence, where the maximum common sub-sequences between transcripts were condensed into nodes and the variable regions connected by edges. Such an approach has been used to generate the Alternative Splicing Gallery (ASG) resource [5]. ECGene [13] provides AS analysis of several genomes including chicken, using a combination of genome-based EST clustering and graph-based transcript assembly procedures. ECGene is directed towards the functional analysis of individual AS genes. Ast [14] has focussed on AS events with evolutionary consequences, while Lee [15] has addressed the issue of exon creation and/or loss of conserved exons. ASTALAVISTA [16] provides a graphical analysis for AS events only in the human genome.

We have used comparative genome analysis to analyze transcripts for each gene in the chicken genome with the data obtained from ENSEMBL database [17], and compared this dataset to all available Alternative Splicing Transcript Database (ASTD) [1,2] genes for human and mouse genomes. Comparing genome sequences to shed light on aspects of human biology and medicine is a modern addition to the established use of other species as models [18].

The chicken represents approximately 310 million years (Myr) of vertebrate evolution, along a distinctly different branch compared to mammalian species, such as the human and mouse genomes [18]. This genome is the first to be sequenced at this particular evolutionary distance from humans, providing examples of the most distant genome-wide orthologues possible [18].

In this study, we have used splicing graphs for comparative transcriptome analysis. To facilitate detailed analysis, we have developed component sub-graphs, called "splicing patterns" which provide a rapid and automatic analytical system for complete dissection of transcript diversity. Our approach has been to use directed acyclic splicing graphs, without a genomic DNA sequence as reference, defining exons as nodes, interconnected by introns as edges, with paths through the splicing graph representing the transcripts. Such a schema was applied to the *Drosophila melanogaster *genome [4], to generate the DEDB data resource. Here, the first transcript served as a reference sequence to generate splicing graphs, with automatic rule-based classification of splicing events. The use of exons and introns as nodes and edges, respectively, has the intuitive advantage of biological interpretation. However, since there is an ambiguity in the selection of the reference transcript, we have further developed this scheme by choosing the most conserved exons as distinct reference exons and all others as variant, creating the Alternative Splicing Graph Server (ASGS) [6] for generating splicing graphs. To minimize any gene structure bias in the input data, comparative genome analysis has been carried out on the orthologous subset of AS genes for the three species. We report the comprehensive analysis of all transcripts of chicken, human and mouse, based on splicing graphs, and identify AS events in these three genomes and their functional significance in terms of gene ontology (GO) classification [19].

## Materials and methods

### Data format and sources

As primary data, the ASGS algorithm [6] uses input lines based on the GFF standard file format [20]. The transcript information for human and mouse genomes was extracted from ASTD [1,2]. Chicken transcript data was directly retrieved from ENSEMBL [17] (release 50).

### Classification of alternative splicing events

**Figure 1:**
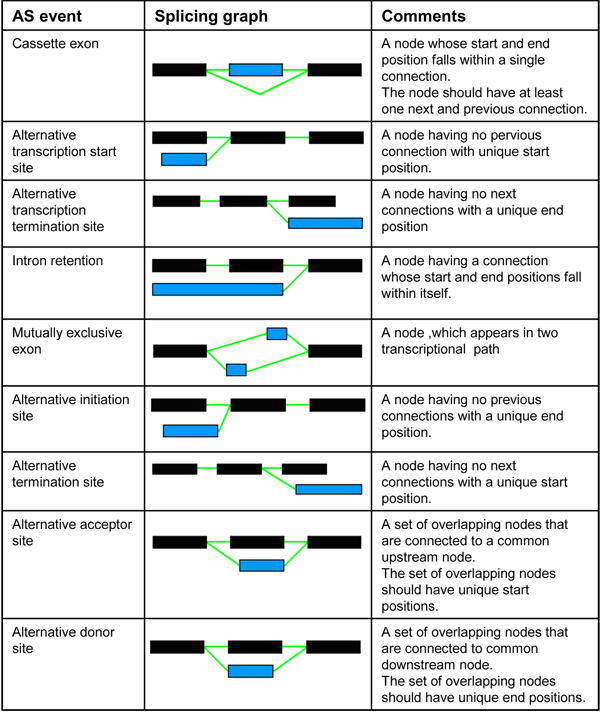
**Generation of alternative splicing (AS) events using splicing patterns. **Distinct exons are shown in black, while variant exons are shown in blue.

We have adopted the classification schema described in DEDB [4], incorporated into the ASGS [6] platform. Rules were derived to detect specific alternative splicing events and these rules are described in Figure 1. Different from ASG [5], we have an enlarged set of AS classification events. Apart from the classical alternative splicing events like cassette exons, intron retention, alternative donor sites and alternative acceptor sites, we have also elected to classify other gene structure events like alternative transcriptional start/termination sites as well as alternative initiation/termination exons. There can be anomalies in the analysis of transcriptional start and termination sites due to sequencing errors. We have also included the ASD [1,2] definition of mutually exclusive exons to this DEDB list of eight AS events and analysed all three genomes for these nine AS events.

### Construction of the splicing graph and systematic splicing pattern detection

Given a set of transcripts for any eukaryotic gene, in terms of the genomic coordinates of the introns and exons of each transcript, there is a step-wise processing to generate the minimum set of component splicing patterns, which is described below.

**Figure 2:**
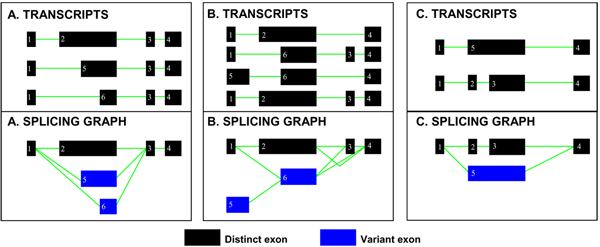
**Classification of exons as distinct and variant**. A. Exon 2 of transcript 1 is selected as the distinct exon, because it is the longest of the three overlapping exons, 2, 5 and 6, at this genomic location. B. Exon 2 is classified as distinct because although both exons 2 and 6 occur twice (number of repeats = 2), exon 2 is longer. C. Exons 2 and 3 are made distinct because exon 5 includes both these exons as well as the intronic region separating them. Thus, exon 5 is classified as variant.

For each transcript or isoforms of a given gene, the genomic coordinates of exons and introns are extracted. All exons are then placed into a new list and sorted based on the genomic position and size. Note that all exons are represented in the standardized sense direction ('+'; 5' to 3'), even if the original transcripts are antisense ('-'; 3' to 5'), for consistency. At every exonic location, the number of times each exon is repeated across all the transcripts is calculated. For each pair of overlapping exons, the one with well-determined boundaries, occurring in the majority of transcripts (repeats >1), is retained as a distinct exon, while the others are classified as variant exons. When comparing two or more exons at the same genomic location, a well-defined boundary is one that is shared among several exons. The longest exon with this well-defined boundary is then considered as the distinct exon and the rest are labelled as variant exons. Figure 2 provides three examples of genes with multiple transcripts, where these classification rules have been applied. In Figure 2A, only exon 2 is different and at this genomic position, there are two shorter exons, 5 and 6, overlapping with exon 2. As each of the exons 2, 5 and 6 occurs only once in all the transcripts, the longest exon is made distinct. In Figure 2B, exon 1 occurs three times at the same genomic location as exon 5, which occurs only once. Using the repeat rule, exon 1 is made distinct, while exon 5 is considered variant. At the same time, exons 2 and 6 each occur twice at the same genomic location. However, as exon 2 is longer and fully contains exon 6, it is made distinct, while exon 6 is classified variant. In Figure 2C, exons 2 and 5 start from the same genomic location, while the end of exon 5 corresponds to that of exon 3. Thus, exons 2 and 3 explicitly define shorter exons contained in exon 5, which also includes the intronic region separating exons 2 and 3. Therefore, the shorter separate exons (2 and 3) are retained as distinct exons, while the longer one (exon 5) is classified as a variant exon. If the shorter exons overlap with other exons, the above rule of repeats and extent is applied prior to labelling them as distinct exons. The above steps are continued till all exons have been sorted as distinct and variant, after which they are sequentially numbered. Distinct and variant exons are then connected, using the intervening intronic regions to generate the splicing graph. The exon pairs in every transcript are then classified as a specific splicing pattern. The exon table, the splicing pattern table and the splicing event table are then generated, for each alternatively spliced gene.

### Decomposition of splicing graphs into splicing patterns

**Figure 3:**
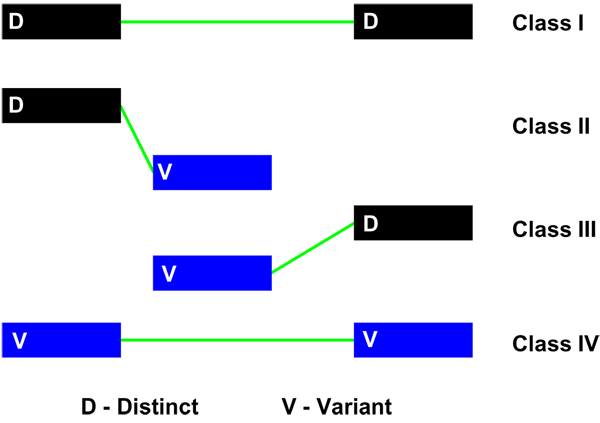
**Classification of inter-exonic connections as splicing patterns**. Four component splicing patterns have been defined, depending on connections between distinct exons (black) and variant exons (blue). Class I refers to connections between two successive distinct exons while Class IV refers to connections between two successive variant exons. Classes II and III depict connections between a distinct exon and a variant exon.

The splicing graph representation provides an intuitive approach to alternative splicing pattern analysis, where gene architecture can be classified using a minimum set of four novel subgraph elements, referred to as splicing patterns. The construction of splicing graphs helps to identify distinct reference (D) and the associated variant (V) exons. The detailed analysis of the relationship of each exon to its successor, designated as a splicing pattern, defines transcript diversity at the fundamental level. The only possible connections available in a splicing graph are distinct-distinct, distinct-variant, variant-distinct and variant-variant. These splicing patterns are labelled as class I (D-D), class II (D-V), class III (V-D) and class IV (V-V) (Figure 3). The splicing graph is colour-coded to represent all distinct (D) exons as black and all the variant (V) exons as blue. AS events (described in Figure 1) can also be represented using splicing patterns.

### Exon and intron size analyses

We have analysed the exon and intron size conservation across the three genomes for the alternatively spliced genes and orthologous AS genes. Basic statistical measures like the mean, median and standard deviation were calculated for all three genomes.

### Exon number analysis

The number of exons per transcript was analysed for the three genomes. The mean, median and standard deviation were calculated.

### Splicing motif analysis

Several genetic diseases are believed to be the result of splice site mutations. Therefore identifying variations in splice site is of utmost importance. The frequencies of the various splice site motifs were computed for chicken and analysed and compared to the splice site information for human and mouse obtained from ASTD. The frequencies were calculated for GT-AG, GC-AG and AT-AC type splice motifs.

### GO analysis

GO Analysis was conducted by using data obtained from Ensembl using the BioMart [21] tool. The text file consisting of Gene Ontologies for orthologous AS genes for chicken human and mouse genomes were reformatted and put through the WEGO [22] tool to obtain the GO plot and the corresponding values for the molecular function, biological process and cellular component.

## Result and discussion

In all, 3901 chicken AS genes have been compared with 16715 human AS genes and 16491 mouse AS genes, suggesting that only 23% of chicken genes are alternatively spliced, compared to the human (68%) and mouse (57%) genomes. This estimate of the extent of AS in the three genomes compares well with the recent AS estimates from the ASAP II database [3] of 22%, 53% and 53% for chicken, human, and mouse genomes, respectively. ECGene [13] AS estimates, however, are 26%, 26% and 31% for chicken, human, and mouse genomes, respectively, for dataset A. The difference in percentage could be because of differences in the methodology used, compared to ASAP II. Brett et al. [9] suggests that the percentage of AS genes in human to be 44% and in mouse to be 33% from all available mRNA/EST data. Ast and co-workers [14] report AS estimates (from EST-based analysis) of 42%, 62% and 57% for chicken, human and mouse, which are normalized to 42%, 43% and 31% respectively. As these results show large variation, we have merely considered the trend in genome-wise AS among species as chicken<mouse< human, which is similar to our results.

To minimize any gene structure bias and to get the best-annotated genes in chicken for analysis, an orthologous set of genes has been used, whereby all AS genes in chicken, which have alternatively spliced orthologous in both human, and mouse were extracted. One-to-one, many-to-many, one-to-many and apparent mappings extracted using BioMart [21], has been used to compile the orthologous genes, collated as the orthologous gene subset (Table 1) of alternatively spliced genes in chicken (2996), human (3091) and mouse (3120). For the orthologous gene subset of AS genes, 17% of chicken genes are alternatively spliced, compared to 12% in human and 11% in mouse. Comparable subsets of AS genes from different organisms were generated using a different approach in the study reported by Brett et al. [9]. Here, the authors generated a random set of 650 mRNA sequences with a coverage of 100,000 ESTs for each organism and reported that AS was consistently around 10% for human, mouse, rat, fly and worm genes. The values that we have obtained for the orthologous AS genes for human and mouse are reminiscent of the overall conclusions reached by Brett et al. [9], lending credibility to our approach of using orthologous AS gene subsets for multi-species comparisons and providing us a reliable method to estimate the extent of AS in chicken.

**Table 1:** Comparison of alternative splicing in human, mouse and chicken genomes.

**Genome**	**Genes**	**Genes with multiples transcripts**	**% of Alternative splicing**	**Transcripts per gene** **(mean ± sd(med))**	**Exon numbers** **per transcript (mean ± sd(med))**	**Exon size (nt)** **(mean ± sd(med))**	**Intron size (nt)** **(mean ± sd(med))**
Chicken	16723	3901	23%	2.4 ± 1.35 (3)	5.3 ± 2.49 (4)	100 ± 105 (50)	3679 ± 3391 (3201)
Human	24573	16715	68%	7.96 ± 8.01 (7)	7.7 ± 5.92 (6)	178 ± 196(89)	5314 ± 4112(4517)
Mouse	28931	16491	57%	6.5 ± 6.01 (5)	6.6 ± 4.15 (5)	159 ± 167(63)	4311 ± 4003(3889)
**Orthologous gene set**							
Chicken	16723	2996	17%	2.45 ± 1.25 (3)	5.1 ± 2.31 (4)	110 ± 102 (49)	3659 ± 3387 (3179)
Human	24573	3091	12%	7.5 ± 7.59 (6)	9.1 ± 7.81 (8)	170 ± 149 (90)	5300 ± 3990(4350)
Mouse	28931	3120	11%	6.2 ± 5.49 (5)	9.0 ± 7.15 (7)	150 ± 153 (75)	4295 ± 3990(3858)

Mean, standard deviation (sd) and median (med) values have been computed for columns 5–8.

### Exon/intron size and exon number analysis

Our results indicate that chicken AS genes (Table 1) are represented by 2.4 transcripts per gene on average, compared to 7.9 and 6.5 transcripts per gene in human and mouse, respectively. We have provided mean, std. deviation and median values for transcripts, exon number, intron sizes and exon sizes, as proposed by Sugnet et al. [23].

General statistical characteristics of the intron-exon structure of eukaryotic genomes are invaluable for understanding the structure and evolution of genes and genomes. Using available gene structure information on ten model organisms, Deutsch and Long [24] estimated that each gene comprises 4.1 exons of 70 nt on average, separated by introns of mean length 1114 nt for chicken. For human their estimates are 4.3 exons of mean length 53 nt separated by introns of mean length 706 nt and for mouse it is 5.0 exons of mean length 51 nt separated by introns of mean length 3413 nt; and 4.4 exons of mean length 52 nt separated by introns of mean length 1321 nt. From this study (Table 1), we find that each chicken transcript comprises close to 5 exons of mean length 100 nt, separated by introns of mean length 3679 nt, while human and mouse transcripts comprise close to 8 and 7 longer exons respectively of mean length 178 nt and 159 nt, separated by longer introns of length 5314 nt and 4311 nt respectively. Thus, chicken AS genes comprise fewer transcripts of fewer and shorter exons, separated by shorter introns, compared to human and mouse AS genes.

These numbers are again similar to those obtained for the orthologous AS gene set, so that while all three transcriptomes are composed of exons of similar size, the introns separating them are shorter in chicken when compared to human and mouse. All further analysis results are based on this orthologous subset.

### Splicing motif analysis

The splicing motif analysis for the complete set of AS genes for chicken, human and mouse genomes yielded consistent values in the three genomes. 99% of the splice site motifs in chicken AS genes were found to be GT-AG (Table 2). The data for the orthologous AS gene set was found to be very similar to that of the complete AS gene set (data not shown).

**Table 2:** Splice site motif analysis for chicken, human and mouse

**Splice site motifs**	**Chicken**	**Human (ASTD)**	**Mouse (ASTD)**
**GT-AG**	99%	99%	98%
**GC-AG**	1%	1%	1%
**AT-AC**	0.07%	0%	0%

### Splicing graphs

We compiled the transcript structure of each multi-transcript gene for all three genomes, using the splicing graph approach described in ASGS [6] and decomposed these splicing graphs into component splicing patterns (described in Materials and Methods). We generated a total of 3901 chicken, 16715 human and 16491 mouse splicing graphs.

### Alternative splicing events and patterns

Based on the splicing patterns, nine AS events (as defined in DEDB [4] and ASD [1,2]) have been identified in the chicken genome and compared to those in human and mouse.

**Figure 4:**
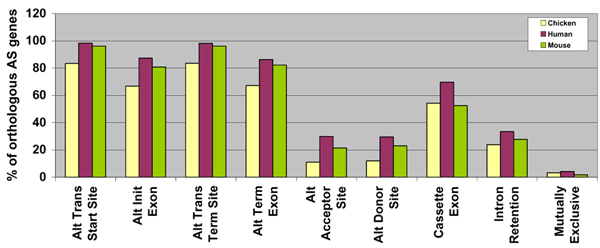
**Distribution of AS events – gene level analysis for chicken, human and mouse orthologous AS genes**. Nine events, described in Figure 1, were used to classify the observed alternative splicing phenomena based on the number of genes that have each of the events using the data in Table 3.

For the gene level analysis (Table 3, Figure 4), the number of orthologous genes showing each AS event was calculated for each of the three genomes. The first four AS event categories in Figure 4 refer to splicing events at the ends of a gene, while the remaining five represent internal events. Of the internal events, the majority of gene represents cassette exons whereas intron retention along with mutually exclusive exons is least represented. Fewer chicken genes show AS events than human or mouse genes. It should be noted that each AS gene contains several events. The orthologous gene set shows similar values for each event and for each genome, compared to the numbers for all AS genes.

**Table 3:** Statistics of AS events for the complete AS genes and the orthologous gene subset (Gene Level Analysis).

**Type of alternative splicing event**	**Chicken (Complete set)**	**Chicken (Orthologous set)**	**Human (Complete set)**	**Human (Orthologous set)**	**Mouse (Complete set)**	**Mouse (Orthologous set)**
Transcriptional Start Site	3068 (79%)	2501 (83%)	16188 (97%)	3039 (98%)	15360 (93%)	2999 (96%)
Alternative Initiation Exons	2053 (53%)	2003 (67%)	13617 (81%)	2702 (87%)	12147 (74%)	2525 (81%)
Transcriptional Termination Site	3129 (80%)	2503 (83%)	16182 (97%)	3037 (98%)	15408 (93%)	3001 (96%)
Alternative Termination Exons	2071 (53%)	2015 (67%)	13658 (82%)	2670 (86%)	12303 (75%)	2566 (82%)
Alternative Acceptor	395 (10%)	332 (11%)	4560 (27%)	924 (30%)	3292 (20%)	670 (21%)
Alternative Donor	405 (10%)	358 (12%)	4616 (28%)	912 (30%)	3305 (20%)	719 (23%)
Cassette Exons	1828 (47%)	1627 (54%)	10392 (62%)	2153 (69%)	7341 (45%)	1639 (52%)
Intron Retention	764 (20%)	716 (23%)	5643 (34%)	1035 (33%)	4412 (27%)	868 (28%)
Mutually Exclusive	106 (3%)	100 (3%)	389 (2%)	128 (4%)	160 (1%)	56 (2%)

Data on nine AS events described in Figure 1 have been compiled.

**Figure 5:**
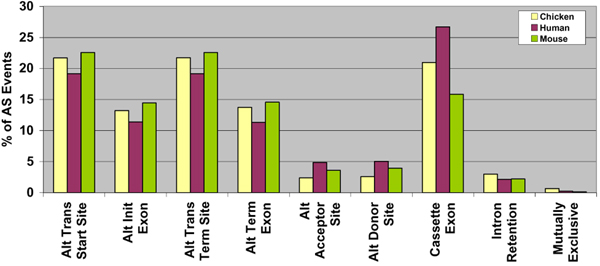
**Distribution of AS events – event level analysis for chicken, human and mouse orthologous AS genes**. Event level analysis calculated the total number of times each of the nine events had taken place in the three genomes based on the values described in Table 4, for the three genomes.

The event level analysis (Table 4, Figure 5) considers the distribution of each event compared to the total number of AS events observed in the orthologous set of the three genomes. The results show considerable conservation in each of the nine AS events for the three species. The first four AS event categories in Figure 5 refer to splicing events at the ends of a gene, while the remaining five represent internal events. Since the end events are subject to sequencing errors, they have not been analysed further, except to state that we observe similar trends in all three species. Exon skipping or cassette exon is found to be the most prevalent AS event in all three species, comprising 21%, 27% and 16% of all AS events in chicken, human and mouse, respectively. On the other hand, intron retention and mutually exclusive exons were the least favoured AS events. Intron retention accounted for only 3% of chicken AS events, compared to 2% in human and 2% in mouse. These values are more conservative than ASD [1,2] reports of 52% cassette exons and 17% intron retention. Mutually exclusive exons are also relatively few, supported by 1% of AS events in chicken, 0.2% in human and mouse.

**Table 4:** Statistics of AS events for the orthologous gene subset (Event Level  Analysis).

**Type of alternative splicing event**	**Chicken**	**Human**	**Mouse**
Transcriptional Start Site	7259 (21%)	22345 (19%)	17240 (23%)
Alternative Initiation Exons	4416 (13%)	13281 (11%)	11019 (14%)
Transcriptional Termination Site	7272 (22%)	22344 (19%)	17325 (23%)
Alternative Termination Exons	4588 (14%)	13205 (11%)	11114 (15%)
Alternative Acceptor	806 (2%)	5696 (5%)	2772 (4%)
Alternative Donor	870 (3%)	5888 (5%)	3012 (4%)
Cassette Exons	6998 (21%)	31158 (27%)	12094 (16%)
Intron Retention	1000 (3%)	2517 (2%)	1701 (2%)
Mutually Exclusive	223 (0.6%)	276 (0.2%)	126 (0.2%)
**Total events**	**33432**	**116710**	**76313**

Overall, from the two sets of analyses, fewer chicken genes show AS events compared to human and mouse, which implies that fewer AS genes in chicken show high transcript diversity as opposed to human and mouse genes.

**Figure 6:**
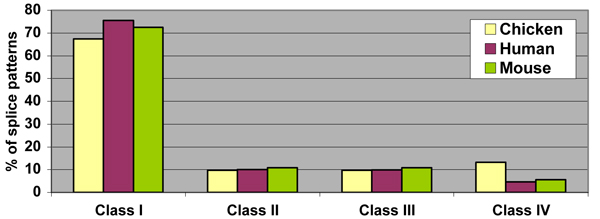
**Splicing pattern distribution in the orthologous chicken, human and mouse AS genes**. Statistics on four components of splicing patterns have been complied, with the transcript diversity index defined as the fraction of all patterns involving variant exons.

To determine the exact nature of the transcript diversity, the splicing pattern analysis was done for the orthologous AS genes by calculating the percentage of the four Classes in the splicing pattern. From the results (Table 5 and Figure 6), in human and mouse genomes, the genome-wise trend in splicing patterns is maintained, with Class I > Class II and Class III > Class IV. However in the chicken genome it was found that Class I > Class IV and Class II > Class III. In all three genomes, Class I patterns (linking two distinct exons) represent the major splicing pattern (chicken: 67%, human: 75%, mouse: 72%). The amount of splicing patterns present is indicative of the extent of transcript diversity within a genome. We define the transcript diversity index (TDI) as the sum of splicing patterns involving variant exons (Classes II, III and IV), which is also (100 – Class I). TDI in chicken is 33% compared to 24% for human and 27% for mouse (Table 5). TDI values show that same trend as estimates of alternative exons from HOLLYWOOD [25] (human: 25% and mouse: 13%) and ASAP II [3] (human: 36% and mouse: 21%), although the methodology for classifying constitutive and alternative exons is somewhat different from the distinct and variant exon classification we have used (described in Methods). TDI represents a quantitative measure of complexity in the transcriptome.

**Table 5:** AS class distribution based on splicing patterns for orthologous chicken, human and mouse AS genes.

**Genome**	**Class I**	**Class II**	**Class III**	**Class IV**	**Total**	**TDI = 100 – % Class I**
Chicken	66433 (67.3%)	9588 (9.7%)	9560 (9.7%)	12992 (13.2%)	98573	33
Human	169419 (75.5%)	22391 (9.9%)	22246 (9.2%)	10220 (4.5%)	224276	24
Mouse	111140 (72.5%)	16720 (10.9%)	16705 (10.9%)	8630 (5.6%)	153195	27

### GO Analysis of orthologous gene sets

**Figure 7:**
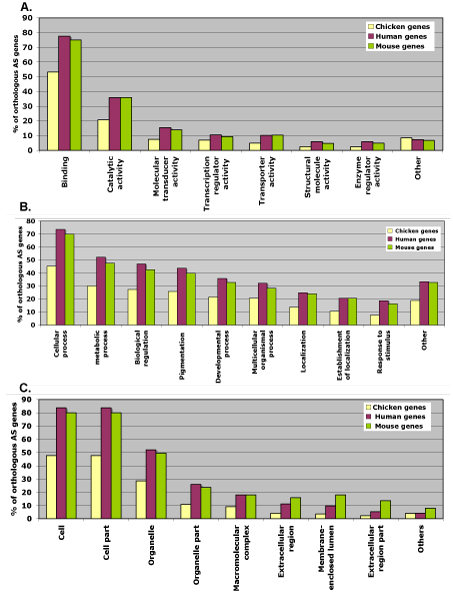
**Occurrence of GO terms in chicken, human and mouse for the orthologous gene subset**. GO terms have been categorized on the basis of A. molecular function, B. biological process and C. cellular component

Gene Ontology (GO) analysis was carried for all three organisms on the orthologous set. The entire GO details were obtained from Ensembl using the BioMart tool [21]. This analysis showed considerably low percentage for chicken as opposed to the human and mouse. In the plot obtained, a considerable drop in functionality was noticed across all the areas for the chicken genome. (Table 6, Figure 7).

**Table 6:** Gene ontology (GO) annotation summary for the orthologous AS gene set.

**A. Molecular Function**	**% Chicken genes**	**% Human genes**	**% Mouse genes**
Binding	53.2	77.6	75.0
Catalytic activity	20.6	35.8	36.0
Molecular transducer activity	7.5	15.4	13.9
Transcription regulator activity	6.9	10.4	9.4
Transporter activity	4.9	10.1	10.3
Structural molecule activity	2.4	5.9	4.7
Enzyme regulator activity	2.3	5.9	4.9
Other	8.5	7.3	6.8
**B. Biological Process**	**% Chicken genes**	**% Human genes**	**% Mouse genes**
Cellular process	45.2	73.6	69.8
metabolic process	30.0	52.1	47.7
Biological regulation	27.4	46.9	42.6
Pigmentation	25.8	43.8	39.9
Developmental process	21.6	35.8	32.7
Multicellular organismal process	20.5	32.2	28.4
Localization	13.7	24.8	24.1
Establishment of localization	10.7	20.7	20.6
Response to stimulus	7.6	18.7	16.4
Other	19	33.3	32.7
**C. Cellular Component**	**% Chicken genes**	**% Human genes**	**% Mouse genes**
Cell	47.8	83.7	79.9
Cell part	47.8	83.7	79.9
Organelle	28.5	52.1	49.5
Organelle part	10.8	25.8	23.6
Macromolecular complex	9.1	18.0	18.0
Extracellular region	4.1	11.1	15.8
Membrane-enclosed lumen	3.5	9.6	18.0
Extracellular region part	2.3	5.2	13.6
Others	4.0	4.1	7.9

## Direction for future study

### Investigation of the tendency of introns to lie in the protein domain boundaries

Having introns near the domain boundaries are thought to allow exon shuffling easily. According to the exon shuffling theory, it is believed that shuffled exons can create functional diversity in novel proteins making the species more adaptive during evolution [26]. The domain boundaries can be analysed as explained in Liu and Grigoriev [26] to determine if there is any tendency for introns to lie near the domain boundaries. We could count the number of introns lying within 10 amino acids of domain boundaries as well as the number of introns falling outside this 10 amino acid region. The expected number of introns to fall within and outside the regions can then be computed and used for a Chi Square Goodness-of-Fit test to determine if the number of introns lying in the domain boundaries deviate from the expected.

An extension to the exon shuffling theory is the splice frame rule, which states that the phases of introns bordering domains tend to match following a successful shuffling event [27]. This allows the exon flanked by the introns to shuffle more easily as the coding frame is retained. Therefore a Chi Square Goodness-of-Fit test can be done to determine if there is a tendency for these flanking introns to be symmetrical. Symmetrical introns are a pair of introns where the intron phase is the same.

## Conclusion

We have developed a novel subgraph-based analytical scheme for comparative transcriptome analysis, using a set of four discrete splicing patterns. Using this methodology we have analysed and compared the transcript diversity present in the entire chicken genome with data available for human and mouse.

This comprehensive study of the chicken transcriptome, showed that 23% of chicken genes undergo alternative splicing compared to 68% and 57% in human and mouse, respectively. Our analyses also showed that chicken AS genes are composed of fewer transcripts when compared to human and mouse. However we noticed that the introns in chicken were of considerably shorter length when compared to human and mouse. We have compared nine different splicing events among chicken, human and mouse genomes. At the event level, the most common AS event was found to be exon skipping and the least common events was intron retention and mutually exclusive exons. Overall, fewer AS chicken genes show high transcript diversity, with predominantly introns linking two variant exons, as opposed to human and mouse genes. We have also defined the transcript diversity index (TDI), for quantifying complexity within the AS genes of any species, as the fraction of splicing patterns involving variant exons. Chicken has a TDI value of 33% compared to 24% in human and 27% mouse, representing the complexity of the transcriptome of that organism.

We have also collected the GO definitions for alternatively spliced genes. The chicken AS genes orthologous to human and mouse also show functional similarity, based on the GO classifications. This work will be continuously updated as more data becomes available, and also extended to other species for a multi-genome comparison.

The analysis of chicken AS genes compared to human and mouse, using the splicing graph approach, has provided a deep understanding of the transcript diversity in chicken, with details of the AS events that occur and their gene ontology classification. Based on the transcript diversity index computed on the available chicken dataset, the chicken transcriptome shows greater transcript diversity than those of human and mouse, characterized by predominantly adjacent variant exons.

## Competing interests

The authors declare that they have no competing interests.

## Authors' contributions

SR conceived the alternative splicing analysis concept for the chicken genome. EC obtained the data and carried out the analysis. EC and SR wrote the paper. All authors approved the manuscript and declare that there is no conflict of interest.
